# RBMS1 Contributes to Early Arteriovenous Fistula Neointimal Remodeling Through Modulation of VSMC Phenotypic Responses

**DOI:** 10.1002/advs.78286

**Published:** 2026-10-11

**Authors:** Zongda Li, Chengwei Zhang, Xinying Yu, Shuang Yao, Yiting Lai, Xiaozhu Sun, Yao Zhao, Xiaoyu Lan, Tingting Hu, Wenjin Li, Zhenzhen Ma, Huimin Ma, Zihan Wang, Haihai Liang, Jundong Jiao

**Affiliations:** ^1^ Department of Nephrology The Second Affiliated Hospital of Harbin Medical University Harbin People's Republic of China; ^2^ Laboratories of Biomedicine‐Pharmaceutics of China, Key Laboratory of Cardiovascular Research, Ministry of Education), College of Pharmacy, Harbin Medical University State Key Laboratory of Frigid Zone Cardiovascular Diseases (SKLFZCD), Department of Pharmacology (State Key Laboratory Province Key Harbin Medical University Harbin People's Republic of China; ^3^ Institute of Nephrology Harbin Medical University Harbin People's Republic of China

**Keywords:** alternative splicing, arteriovenous fistula, neointimal Hyperplasia

## Abstract

Arteriovenous fistula (AVF) creation initiates stage‐dependent remodeling of the venous outflow tract. Neointimal hyperplasia is a common component of this response, but its post‐transcriptional regulation remains unclear. Here, analyses of human AVF datasets and tissue specimens characterized RNA‐binding motif single‐stranded interacting protein 1 (RBMS1) expression in postoperative AVFs, whereas a day‐14 mouse AVF model and cultured vascular smooth muscle cells (VSMCs) were used to investigate its role in early neointimal remodeling. Smooth muscle‐targeted RBMS1 overexpression aggravated neointimal formation, whereas conditional RBMS1 deletion attenuated it in murine AVFs. In cultured VSMCs, RBMS1 overexpression enhanced proliferation, migration, and the transition from contractile to synthetic marker expression, whereas RBMS1 silencing produced the opposite effects. Mechanistically, RBMS1 bound myelin protein zero‐like 1 (MPZL1) mRNA and promoted exon 5 skipping, generating the MPZL1‐Δ5 isoform. MPZL1‐Δ5 overexpression reproduced selected RBMS1‐associated VSMC responses and increased neointimal hyperplasia in vivo, including in RBMS1‐deficient mice. MPZL1‐Δ5 further interacted with GTPase‐activating protein SH3 domain‐binding protein 1 (G3BP1) and enhanced protein kinase B (AKT) activation, whereas G3BP1 silencing attenuated RBMS1‐ or MPZL1‐Δ5‐induced VSMC responses. Together, these findings identify the RBMS1–MPZL1‐Δ5–G3BP1–AKT pathway as an important VSMC‐associated mechanism contributing to neointimal remodeling during the early postoperative phase of experimental AVFs.

## Graphical abstract

Postoperative arteriovenous fistulas exhibit increased RBMS1 in vascular smooth muscle cells (VSMCs). In experimental AVFs, RBMS1 overexpression aggravates, whereas deletion attenuates, neointimal hyperplasia. RBMS1 binds MPZL1 mRNA and promotes exon 5 skipping to generate MPZL1‐Δ5, which interacts with G3BP1 to activate AKT, driving VSMC proliferation, migration, and phenotypic switching. This pathway defines a mechanism underlying early experimental AVF remodeling.

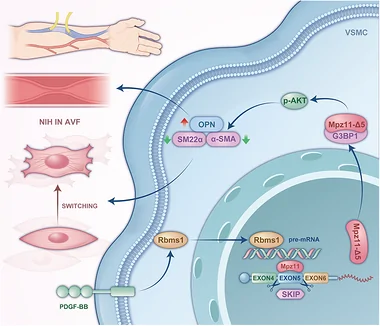

## 1 Introduction

Chronic kidney disease (CKD) represents a significant global public health challenge and may progress to end‐stage kidney disease (ESKD), for which kidney replacement therapy is required. Hemodialysis is the predominant kidney replacement modality for patients with ESKD [1]. Therefore, the establishment of reliable vascular access is critical for effective long‐term hemodialysis. Arteriovenous fistula (AVF) is the preferred long‐term vascular access according to clinical guidelines, due to its superior patency, lower risk of infection and thrombosis, and association with reduced patient mortality [2]. Following AVF creation, the venous wall undergoes concurrent inward and outward remodeling, and access function reflects the resulting changes in lumen geometry and wall properties [3]. NIH is a common histopathological feature that may contribute to luminal narrowing. However, intimal thickening is frequently present in pre‐access veins, and clinical studies have not established a consistent relationship between its extent and subsequent maturation or patency [4]. NIH should therefore be viewed as an important pathological process within AVF remodeling, whose functional consequences are shaped by the accompanying structural, mechanical, inflammatory, and thrombotic changes in the venous wall [5].

Human studies increasingly support AVF remodeling as a multicellular and stage‐specific process involving coordinated changes across vascular, stromal, and immune cell populations [6]. Human pre‐access veins contain substantial fibroblast and myofibroblast populations that contribute to extracellular matrix production, vascular remodeling, and tissue homeostasis. Recent single‐cell studies further indicate that fibroblasts and macrophages are more abundant than medial vascular smooth muscle cells (VSMC) in human AVFs. Early remodeling involves monocyte infiltration and reprogramming of fibroblasts and myofibroblasts toward reparative states, whereas later remodeling is dominated by extracellular matrix‐producing stromal cells. Failed AVFs exhibit persistent inflammation, activated macrophages, and hyperactivated fibroblast and myofibroblast states. Together, these findings indicate that AVF maturation and failure involve interactions among multiple cellular states [7].

Within this multicellular context, VSMCs remain important contributors to AVF‐associated neointimal remodeling [8, 9, 10]. Differentiated VSMCs may contribute to venous wall thickening and arterialization, whereas phenotypically modulated VSMCs can participate in neointimal lesion formation. Our previous studies have shown that METTL3‐mediated N6‐methyladenosine (m6A) modification and the LncDACH1 / protein kinase B (AKT) signaling axis enhance VSMC migration and phenotypic modulation, thereby contributing to NIH progression [11, 12]. Nevertheless, the post‐transcriptional mechanisms regulating VSMC responses during AVF‐associated neointimal remodeling remain incompletely understood. We therefore investigated RBMS1 as a candidate regulator of VSMC‐associated remodeling within this multicellular environment.

Alternative splicing (AS) is a critical regulatory mechanism in gene expression that enables the generation of mature mRNAs with distinct exon combinations from a single precursor, thereby significantly expanding transcriptomic diversity and functional potential [13]. Advances in RNA sequencing technologies, coupled with the development of computational tools, have facilitated transcriptome‐wide analyses of AS with unprecedented resolution [14, 15]. Increasing evidence highlights that dysregulated AS contributes to the pathogenesis of numerous cardiovascular diseases [16]. Notably, aberrant expression of RNA‐binding proteins (RBPs) has been identified as a key factor driving AS dysregulation and disease progression. RBPs represent a large family of proteins that bind RNA through specific sequence motifs or structural elements [17]. Most RBPs contain conserved domains, such as RNA recognition motifs (RRMs), ribonucleoprotein motifs, or K homology domains, which mediate selective RNA binding and regulate RNA splicing, stability, localization, and translation [18, 19]. Emerging evidence suggests that RBPs play pivotal roles in cardiovascular pathologies, including coronary atherosclerosis, cardiac fibrosis, and pulmonary arterial hypertension [20, 21].

RNA‐binding motif single‐stranded interacting protein 1 (RBMS1) is a key member of the RBP family. Initially identified as a single‐stranded DNA‐binding protein associated with the c‐Myc gene, RBMS1 has been shown to suppress the transcription of target genes [22, 23]. It contains two RRM domains, providing the structural foundation for its RNA‐binding ability and its role in regulating AS [22]. RBMS1 and its encoded protein have been implicated in the regulation of DNA replication, apoptosis, and cell growth, as well as in pathological processes such as cardiovascular disease, obesity, and tumorigenesis [24, 25, 26, 27, 28]. However, the physiological and pathological roles of RBMS1 in AVF‐associated NIH remain poorly understood.

In this study, we examined RBMS1 expression in human postoperative AVF tissues and investigated its mechanistic role in early experimental AVF neointimal remodeling. RBMS1 was identified as a splicing factor that binds myelin protein zero‐like 1 (MPZL1) mRNA and promotes the generation of an exon 5‐deleted isoform, MPZL1‐Δ5. This isoform interacts with GTPase‐activating protein SH3 domain‐binding protein 1 (G3BP1), enhances AKT signaling, and promotes VSMC‐associated neointimal formation in experimental AVFs. These findings identify an RBMS1‐dependent splicing mechanism that contributes to the neointimal component of early AVF remodeling within a broader multicellular process.

## 2 Method

### 2.1 Data Access

Detailed descriptions of all experimental procedures and materials are provided in the Supplemental Material. The data supporting this study are available from the corresponding author upon reasonable request. The transcriptomic datasets reanalyzed in this study are deposited in the Gene Expression Omnibus (GEO) under accession numbers GSE244317, GSE283628, GSE305423, and GSE220796, and the RNA immunoprecipitation sequencing data generated in this study are deposited under GSE327537. GSE244317 comprises bulk poly(A) RNA‐sequencing data from six AVF outflow inferior vena cava samples and six sham inferior vena cava samples collected from 10‐week‐old C57BL/6J mice on postoperative day 7, with three male and three female mice per group [29]. All AVFs were patent at tissue collection. Gene function annotation and pathway enrichment analyses were conducted using the Gene Ontology (GO) and Kyoto Encyclopedia of Genes and Genomes (KEGG) databases. Results from the enrichment analyses were visualized using the ggplot2 package, and enriched GO terms and KEGG pathways were presented as bubble plots. GSE283628 contains human spatial transcriptomic data and human and murine single‐cell RNA‐sequencing data. Human pre‐access controls were surplus cephalic veins collected during AVF creation, whereas postoperative AVF tissues were obtained from different kidney transplant recipients undergoing AVF removal. The human single‐cell dataset included 12 617 pre‐access vein cells and 9277 postoperative AVF cells, whereas the murine component included 11 922 cells from age‐matched jugular veins and 8385 cells from cervical end‐to‐side AVFs. GSE283628 was used to assess the cellular and spatial distribution of RBMS1 during postoperative AVF remodeling [30]. GSE305423 comprises 21 human single‐cell RNA‐sequencing libraries from 20 patients, including six pre‐access vein libraries, three early‐AVF libraries from two patients, six mature‐AVF libraries, and six failed‐AVF libraries [7]. GSE220796 contains paired bulk RNA‐sequencing data from pre‐access basilic veins and postoperative juxta‐anastomotic AVF tissues collected 7–16 weeks after AVF creation. The present analysis included 20 complete pairs deposited within GSE220796, comprising nine matured and 11 failed AVF pairs. In the original study, maturation failure was defined as failure to achieve a luminal diameter of at least 6 mm, requiring a modified second‐stage procedure rather than standard transposition. One postoperative AVF sample whose paired pre‐access vein was deposited in GSE119296 was excluded from the analysis [31].

### 2.2 Human Samples

Human sample collection was conducted at the Blood Purification Center of the Second Affiliated Hospital of Harbin Medical University between May 2024 and May 2025. The study protocol was approved by the Ethics Committee of the Second Affiliated Hospital of Harbin Medical University, and all procedures involving human participants were performed in accordance with the Declaration of Helsinki. Written informed consent was obtained from all participants before surgery. Pre‐access vein samples were collected from patients with stage 5 chronic kidney disease undergoing AVF creation. Postoperative AVF samples were obtained from discarded fistula segments removed during clinically indicated surgical revision or reconstruction, generally after prolonged dialysis exposure. The pre‐access vein and AVF samples were unpaired and were obtained from different patients, with four independent samples included in each group.

Obtaining paired pre‐access and postoperative AVF tissues from the same patient is clinically difficult because AVF segments are not routinely excised and dysfunctional AVFs are now commonly treated by percutaneous transluminal angioplasty. The available postoperative AVF specimens were therefore obtained only when surgical excision was clinically indicated. Accordingly, these institutional samples were used to assess postoperative or chronic venous remodeling and were not designed as an outcome‐matched cohort for distinguishing mature from failed AVFs. To reduce potential sampling and processing bias, all samples were collected using consistent procedures and processed under standardized conditions. Available baseline clinical characteristics are summarized in Table S1. Given the small and unpaired institutional cohort, baseline characteristics were summarized primarily for descriptive characterization; non‐significant P values were interpreted cautiously and were not considered evidence of group equivalence or absence of residual confounding. Samples used for histological and immunostaining analyses were fixed in formalin and embedded in paraffin, whereas samples used for RNA and protein analyses were snap‐frozen and stored at −80°C.

### 2.3 Animal Models

All animal experiments were approved by the Animal Ethics Committee of the Second Affiliated Hospital of Harbin Medical University. Mice were housed under standard conditions with a 12‐h light‐dark cycle, controlled temperature and humidity, and ad libitum access to food and water, and were acclimatized for at least one week before experimentation. Anesthesia was induced with isoflurane, after which mice were placed in the supine position, and the inferior vena cava and abdominal aorta were exposed. In the AVF group, an arteriovenous fistula was created by simultaneous puncture of the distal inferior vena cava and abdominal aorta using a 25‐gauge needle, whereas control mice underwent puncture of the abdominal aorta only. Successful fistula creation was confirmed intraoperatively by observing arterialized blood flow within the inferior vena cava. Postoperatively, mice were kept warm and received buprenorphine analgesia every 12 h.

Fourteen days after surgery, mice were euthanized by CO_2_ inhalation followed by cervical dislocation as a secondary method to ensure death. Death was confirmed by the absence of respiration and reflex responses before tissue collection. The venous outflow segment of the inferior vena cava surrounding the fistula region was harvested from AVF mice, and the corresponding inferior vena cava segment at the same anatomical level was collected from sham‐operated mice. For histological and immunostaining analyses, tissues were fixed, embedded, and transversely sectioned. For quantitative reverse transcription PCR (qRT‐PCR) and Western blotting, tissues from the same anatomical segment were snap‐frozen and stored until further analysis. In this model, the primary histological endpoint was neointimal hyperplasia in the venous outflow segment rather than flow‐limiting stenosis.

### 2.4 Generation of Smooth Muscle Cell‐Specific RBMS1 Conditional Knockout Mice

To generate smooth muscle cell‐specific RBMS1 conditional knockout mice, mice carrying floxed alleles of the RBMS1 gene were crossed with smooth muscle myosin heavy chain (SM‐MHC)–CreERT2 transgenic mice (Cyagen Biosciences, China). Adult mice carrying both the floxed alleles and the Cre recombinase transgene were intraperitoneally injected with tamoxifen (Cat:IT17201, Solarbio, China) at a dose of 80 mg/kg body weight once daily for five consecutive days to induce gene recombination. Littermate controls lacking the Cre recombinase transgene received tamoxifen injections using the same dosing regimen. Genomic DNA extracted from tail tissue was analyzed by polymerase chain reaction to confirm the genotypes.

### 2.5 Generation of RBMS1 Overexpression Mice Using Adeno‐Associated Virus

For smooth muscle cell‐directed overexpression of RBMS1 (GeneChem, China) or the exon 5‐deleted isoform of MPZL1 (MPZL1‐Δ5; Obio Technology, China), AAV serotype 9 vectors driven by the SM22α promoter were used; the matched negative‐control vector encoded GFP as a reporter. For each mouse, 10 µL of viral suspension at a titer of 4 × 10^12 vg/mL, corresponding to a total dose of 4 × 10^10 vg per mouse, was mixed with 50 µL of Pluronic F127 hydrogel (Cat. No. P2443, Sigma‐Aldrich, USA). During AVF surgery, the resulting 60 µL mixture was applied once to the adventitial surface of the inferior vena cava venous outflow segment surrounding the fistula.

### 2.6 Cell Culture and Treatment

Mouse aortic vascular smooth muscle cells were obtained from a commercial supplier and cultured in high‐glucose Dulbecco's modified Eagle medium (DMEM) (Cat#11965092, Gibco, USA) supplemented with 10% fetal bovine serum and 1% penicillin‐streptomycin at 37°C in a humidified atmosphere with 5% CO2. To induce migration, proliferation, and phenotypic switching, VSMCs were treated with platelet‐derived growth factor‐BB (PDGF‐BB) (Cat#HY‐P7087, MedChemExpress, USA) for 48 h before protein analysis. Human embryonic kidney 293T cells were cultured under the same standard culture conditions.

### 2.7 Plasmid Construction and Small Interfering Ribonucleic Acid Preparation

Expression plasmids encoding RBMS1(GenePharma, China) and its truncation mutants, as well as minigene reporter constructs, were generated by commercial vendors. Plasmids encoding the exon 5‐deleted isoform of MPZL1(Obio Technology, China) were also constructed. The minigene reporter plasmid used in this study was constructed by GeneChem (China). Small interfering RNAs (siRNAs) targeting RBMS1(Sangon Biotech, China) and G3BP1(Sangon Biotech, China), along with negative control RNAs, were synthesized commercially. Transfections were performed using a lipid‐based transfection reagent(Cat#C0533, Beyotime, China).

### 2.8 Statistical Analysis

Experimental quantitative data are presented as mean ± standard error of the mean (SEM) unless otherwise indicated, and clinical baseline characteristics are presented as mean ± standard deviation (SD) or as counts and percentages. The value of n represents biologically independent samples, animals, cells, tissue spots, or independent experiments, as specified in the figure and table legends. qRT‐PCR data were normalized to glyceraldehyde‐3‐phosphate dehydrogenase (GAPDH) and analyzed using the 2−ΔΔCt method, and Western blot data were normalized to the corresponding loading controls. No data transformation was applied unless otherwise stated, and outliers were excluded only for predefined technical reasons. Statistical analyses were performed using GraphPad Prism software version 9.5.1. For two‐group comparisons, two‐sided unpaired Student's *t*‐test or Welch's corrected *t*‐test was used according to variance equality. For multiple‐group comparisons, two‐sided one‐way analysis of variance (ANOVA) followed by Tukey's multiple‐comparison test was used. Categorical clinical variables were analyzed using Fisher's exact test or chi‐square test, as appropriate. Exact P values are reported where applicable, and a two‐sided P < 0.05 was considered statistically significant.

## 3 Result

### 3.1 RBMS1 Expression Is Increased in Postoperative human AVFs and During Early Experimental Neointimal Remodeling

AS analysis of the GSE244317 transcriptome dataset was performed using replicate Multivariate Analysis of Transcript Splicing (rMATS), identifying five major types of AS events (Figure 1A), with exon skipping (ES) being the most prevalent (Figure 1B,C). GO enrichment analysis of all ES events revealed significant associations with actin binding, GTPase regulator activity, small GTPase binding, single‐stranded RNA binding, and regulatory RNA binding (Figure 1D).

**FIGURE 1:**
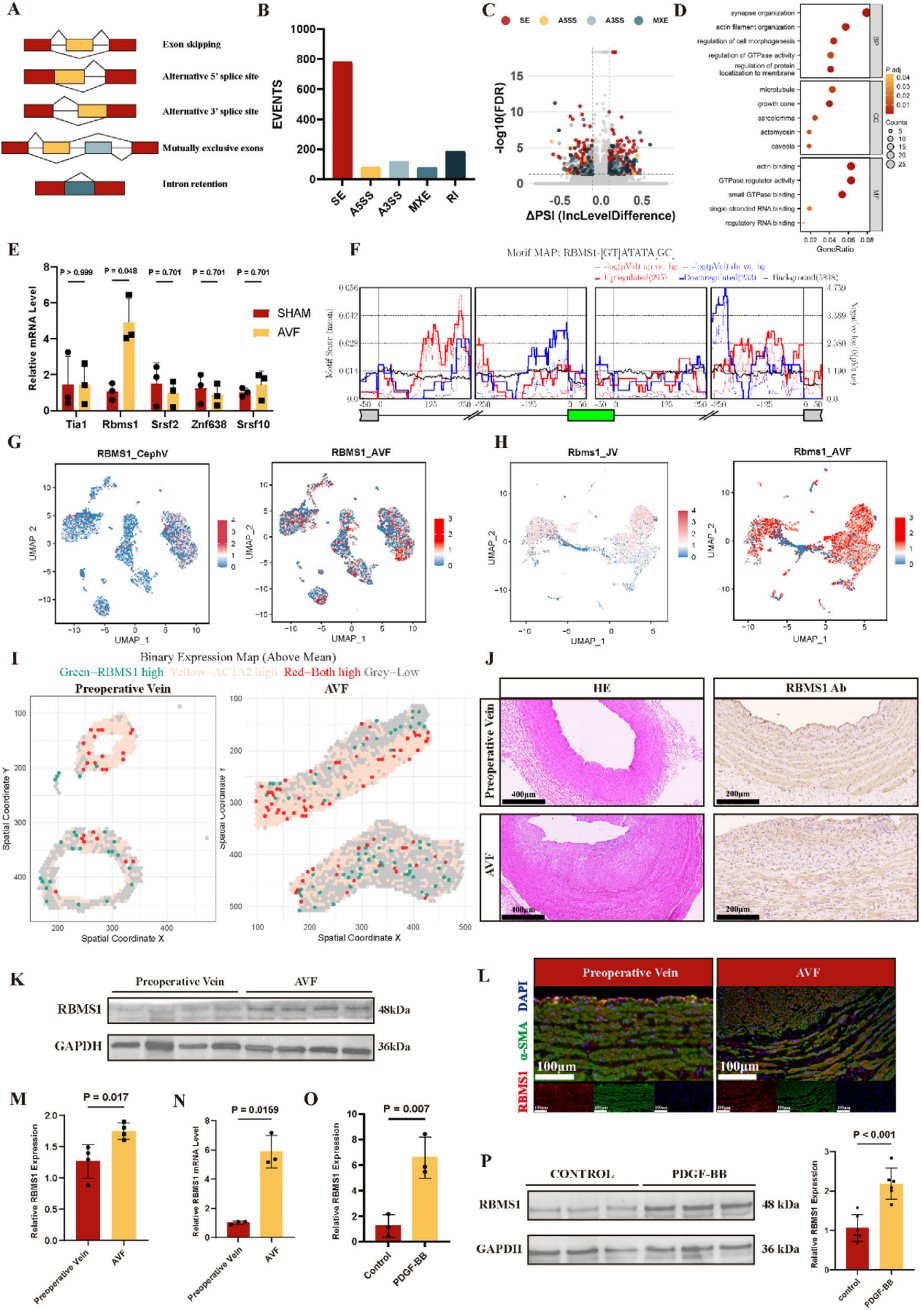
Increased RBMS1 expression in postoperative human AVF tissues and during early experimental AVF remodeling. (A) Schematic diagram illustrating different types of alternative splicing events. (B, C) Statistical summary and volcano plot of alternative splicing events identified in the transcriptomic sequencing of the sham and AVF mouse groups. (D) Bubble plot of enrichment analysis for exon skipping events. (E) In the mouse AVF model, RBMS1 mRNA expression was significantly higher than in sham controls (n = 3 per group). (F) Motif maps of RBMS1. (G) Uniform manifold approximation and projection (UMAP) plot showing RBMS1 expression in pre‐access veins from patients with CKD and postoperative AVF tissues resected from kidney transplant recipients in the GSE283628 single‐cell RNA sequencing dataset, with pre‐access vein cells n = 12 617; AVF n = 9277. (H) UMAP plot showing RBMS1 expression in mouse sham venous tissues and AVF venous outflow tissues, with mouse sham venous cells n = 11 922; AVF n = 8385. (I) Scatter pie plot displaying the expression levels of RBMS1 and α‐SMA at each spot (cephalic veins, n = 186 or 313; venous segments of AVFs, n = 892 or 933). (J) Representative HE staining images and immunohistochemical staining results of RBMS1 in Pre‐access veins and AVF veins from patients with CKD (n = 4 per group); (K, M) Western blot analysis of RBMS1 protein expression relative to GAPDH in pre‐access veins and AVF tissues from patients with CKD (n = 4 per group). (L) Representative immunofluorescence images showing DAPI, α‐SMA, RBMS1, and merged signals in pre‐access veins and postoperative AVF tissue segments from patients with CKD (n = 4 per group). Scale bar, 100 µm. (N) RBMS1 mRNA expression was significantly elevated in AVF veins compared with pre‐access veins from patients with CKD (n = 3 per group). (O) In the model of dedifferentiated vascular smooth muscle cells induced by PDGF‐BB, RBMS1 mRNA was significantly upregulated (n = 3 per group). (P) In the model of dedifferentiated vascular smooth muscle cells induced by PDGF‐BB, RBMS1 protein expression was significantly elevated (n = 5 per group). For single‐cell RNA‐seq panels, n indicates the number of cells analyzed. For spatial transcriptomic panels, n indicates the number of tissue spots. For human tissue validation and mouse tissue experiments, n indicates biologically independent tissue samples or animals. For in vitro VSMC experiments, n indicates independent biological replicates. Data are presented as mean ± standard error of the mean (SEM). Statistical significance was determined by Student's *t*‐test or Welch's test (E, M, O, P). DAPI indicates 4′,6‐diamidino‐2‐phenylindole, used for nuclear staining.

To investigate the regulatory mechanisms underlying ES events, major splicing‐related motifs and their corresponding RBPs were analyzed using the RNA Map Analysis and Plotting Server (rMAPS). The top five RBPs with statistically significant P values were selected for validation in a murine AVF model. PCR revealed that RBMS1 exhibited the most pronounced expression change among these candidates (Figure 1E), and its corresponding motif map was subsequently generated (Figure 1F).

Analysis of single‐cell RNA‐sequencing data from GSE283628 showed more prominent RBMS1 expression in postoperative human AVF tissues resected from kidney transplant recipients than in pre‐access veins from patients with CKD (Figure 1G). A similar increase was observed in the corresponding murine AVF samples (Figure 1H). Spatial transcriptomic analysis further showed increased colocalization of RBMS1 and α‐SMA signals in human AVF tissues (Figure 1I). Because the human AVF specimens in GSE283628 were obtained after kidney transplantation, these findings were interpreted as evidence of postoperative or chronic venous remodeling rather than as evidence of biological events specifically determining maturation outcome.

To define the cellular distribution of RBMS1 within the multicellular human AVF landscape, we analyzed nine major cell populations in GSE305423. RBMS1‐positive cells were detected across vascular, stromal, and immune populations. Among these populations, the VSMC group showed the highest within‐cell‐type RBMS1‐positive fraction, with RBMS1 detected in 65.4% of VSMCs (5336/8160). VSMCs accounted for 12.6% of all RBMS1‐positive cells (5336/42 230) (Figure S1A,B). These findings identified VSMCs as an important cellular context for RBMS1 expression within the multicellular human AVF environment and provided a human‐data‐supported rationale for the subsequent VSMC‐focused mechanistic investigation.

We next examined RBMS1 expression across the clinical sample groups represented in GSE305423. At the sample‐library level, pseudobulk RBMS1 expression in VSMCs differed overall among the pre‐access vein, early‐remodeling AVF, mature AVF, and failed AVF groups (P = 0.011). Median expression was descriptively higher in each postoperative group than in the pre‐access vein group, with the highest values observed during early remodeling (Figure S1C,D). In the independent paired GSE220796 dataset, RBMS1 expression increased after AVF creation in both the matured AVF group (9 pairs, P = 0.0078) and the failed AVF group (11 pairs, P = 0.0020) (Figure S1E). These findings showed that postoperative RBMS1 upregulation occurred across multiple AVF clinical states and was not restricted to maturation failure.

To assess RBMS1 expression in an independent institutional set of human postoperative AVF tissues, unpaired AVF and pre‐access vein samples were examined using HE staining, immunohistochemistry, and immunofluorescence. RBMS1 immunoreactivity was detected in vascular smooth muscle and colocalized with α‐SMA (Figure 1J,L). qRT‐PCR and Western blot analyses further showed higher RBMS1 expression in postoperative AVF tissues than in pre‐access veins from patients with CKD (Figure 1K,M,N). Because these AVF specimens were obtained during late clinically indicated surgical procedures rather than during the early maturation interval, these findings were used as evidence of postoperative venous remodeling and not as evidence of an outcome‐specific mechanism of maturation failure.

In the murine AVF remodeling model, HE staining, immunohistochemistry, and immunofluorescence showed RBMS1 expression in VSMCs within AVF tissues with neointimal hyperplasia and its co‐localization with α‐SMA (Figure S2A–C). Western blot analyses further confirmed a significant increase in RBMS1 expression in the murine AVF model (Figure S2D).

Additionally, RBMS1 expression was markedly upregulated in PDGF‐BB‐treated VSMCs (Figure 1O,P). Together, these single‐cell, spatial transcriptomic, tissue, and in vitro findings consistently linked RBMS1 upregulation to VSMCs during AVF remodeling. These observations suggested that RBMS1 may have an important role in regulating VSMC phenotypic responses during AVF‐associated neointimal remodeling.

### 3.2 RBMS1 Overexpression Enhances VSMC Proliferation, Migration, and Phenotypic Switching and Exacerbates AVF‐associated Neointimal Hyperplasia

To further investigate the role of RBMS1 in VSMCs, an RBMS1 overexpression plasmid was constructed and validated. Following transfection into VSMCs, both RBMS1 mRNA and protein levels were significantly elevated (Figure S3A,B). Counting Kit‐8 (CCK‐8) assays demonstrated that RBMS1 overexpression significantly promoted VSMC proliferation (Figure S3C). Wound healing and Transwell assays further confirmed that RBMS1 overexpression enhanced VSMC migration (Figure S3D,E).

Additionally, RBMS1 overexpression was associated with VSMC phenotypic marker changes, as evidenced by decreased α‐SMA and smooth muscle protein 22 alpha (SM22α) expression and increased osteopontin (OPN) expression (Figure S3F).

To assess the impact of RBMS1 overexpression on AVF‐associated NIH in vivo, a smooth muscle cell‐specific RBMS1 overexpression mouse model was generated using AAV (Figure 2A). Immunofluorescence and Western blot analyses confirmed that AAV‐mediated RBMS1 overexpression significantly increased RBMS1 levels in vascular smooth muscle compared to the negative control group, while AAV‐NC carrying green fluorescent protein (GFP) was co‐transduced to validate the efficiency of the delivery method (Figure 2B,C).

**FIGURE 2:**
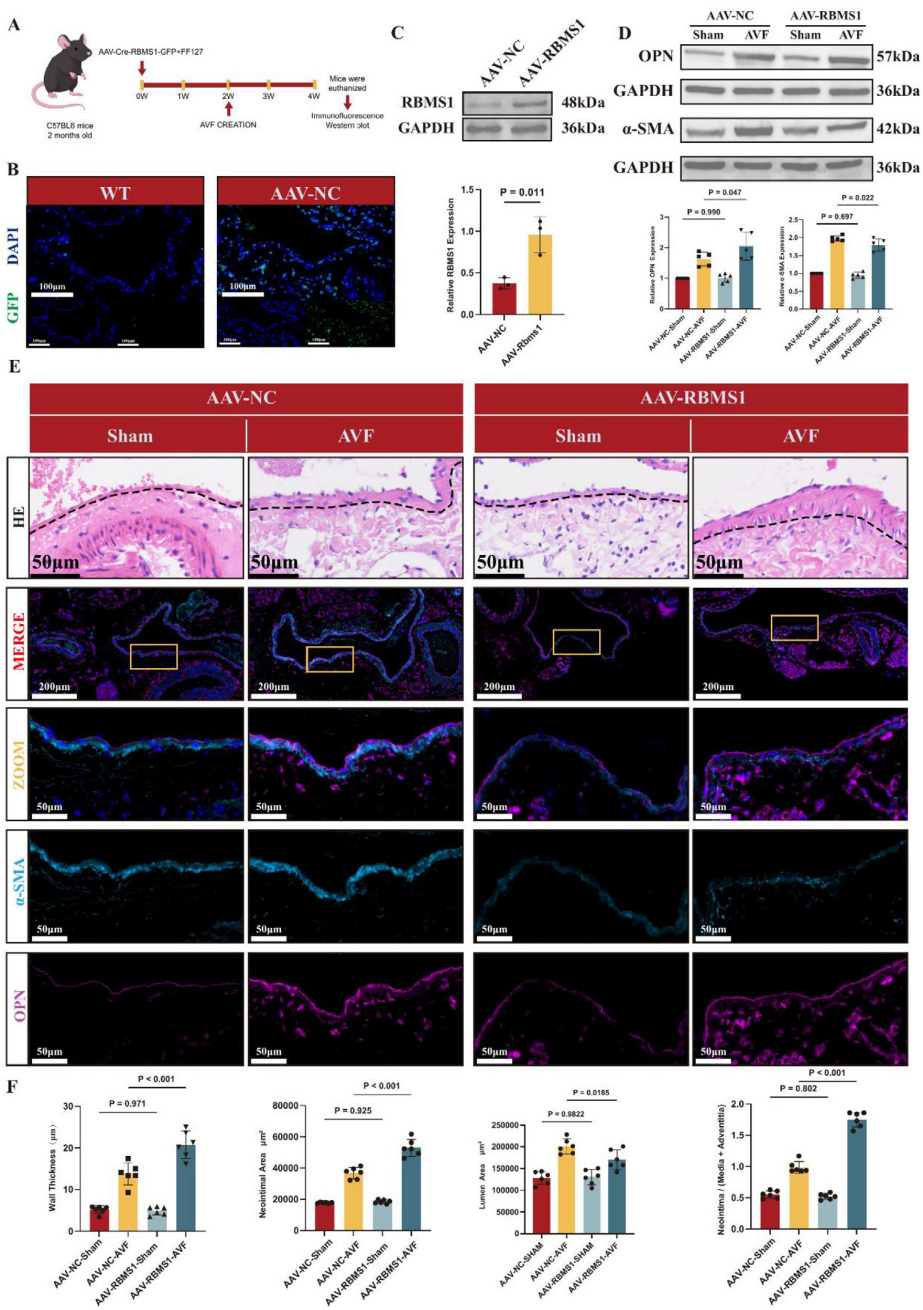
RBMS1 overexpression exacerbates AVF‐associated neointimal hyperplasia. (A) Schematic of the AAV‐RBMS1 mouse model. (B) Immunofluorescence staining showing the efficiency of RBMS1 overexpression in AAV‐RBMS1 mice. (C) Representative Western blot images showing RBMS1 protein expression, with quantification of its relative levels (n = 3). (D) Representative Western blot images showing OPN and α‐SMA protein expression, with quantification of their relative levels (n = 5). (E) HE staining showing neointimal morphology in AAV‐RBMS1 and AAV‐NC mice under sham and AVF conditions (n = 6; original magnification ×40; scale bar = 50 µm). Immunofluorescence staining showing differences in α‐SMA and OPN expression between the two groups (n = 6; scale bar = 50 µm). (F) Morphometric analysis of neointimal hyperplasia in AAV‐RBMS1 and AAV‐NC mice with or without AVF creation (n = 6 per group). n represents biologically independent samples. Data are presented as mean ± SEM. Statistical significance was determined using Student's *t*‐test (C) or two‐sided one‐way ANOVA followed by Tukey's multiple comparison test (D, F).

Histological assessment and neointimal thickness measurements were performed on the venous outflow segment of the inferior vena cava from mice with or without AVF creation. In the sham‐operated group, no significant difference in intimal thickness was observed between AAV‐RBMS1 and AAV‐NC mice (Figure 2E,F). However, in the AVF group, AAV‐RBMS1‐AVF mice exhibited significantly aggravated NIH (Figure 2E,F).

Moreover, analysis of VSMC phenotypic markers revealed no significant differences between AAV‐RBMS1 and control mice in the sham group. In contrast, in the AVF group, RBMS1 overexpression further promoted VSMC phenotypic switching, as indicated by the downregulation of α‐SMA and upregulation of OPN (Figure 2D,E). These results suggest that RBMS1 overexpression is associated with enhanced VSMC phenotypic modulation and increased neointimal thickness in the venous outflow segment in this AVF remodeling model.

### 3.3 RBMS1 Knockdown Suppresses VSMC Proliferation, Migration, and Phenotypic Switching

RBMS1 knockdown in VSMCs was established and validated using siRNA. Compared to control siRNA, transfection with si‐RBMS1 significantly reduced RBMS1 mRNA and protein levels (Figure S4A,B). Wound healing and Transwell assays demonstrated that RBMS1 knockdown significantly inhibited VSMC migration (Figure S4C–F). Additionally, CCK‐8 assays showed a marked reduction in VSMC proliferation upon RBMS1 knockdown (Figure S4G).

Upon confirming dedifferentiation by decreased α‐SMA and SM22α and increased OPN expression, RBMS1 silencing in dedifferentiated VSMCs partially reversed the phenotypic changes, as indicated by the partial restoration of α‐SMA and SM22α and suppression of OPN upregulation (Figure S4H). These results suggest that RBMS1 knockdown suppresses VSMC proliferative, migratory, and phenotypic responses under PDGF‐BB stimulation.

To evaluate the effect of RBMS1 deletion on AVF‐associated NIH in vivo, RBMS1 conditional knockout (cKO) mice were generated using the loxP/Cre system (Figure 3A), with genotypes confirmed by tail DNA analysis (Figure 3B). RBMS1 protein levels in vascular tissues were significantly reduced in RBMS1‐cKO mice compared to controls (Figure 3C).

**FIGURE 3:**
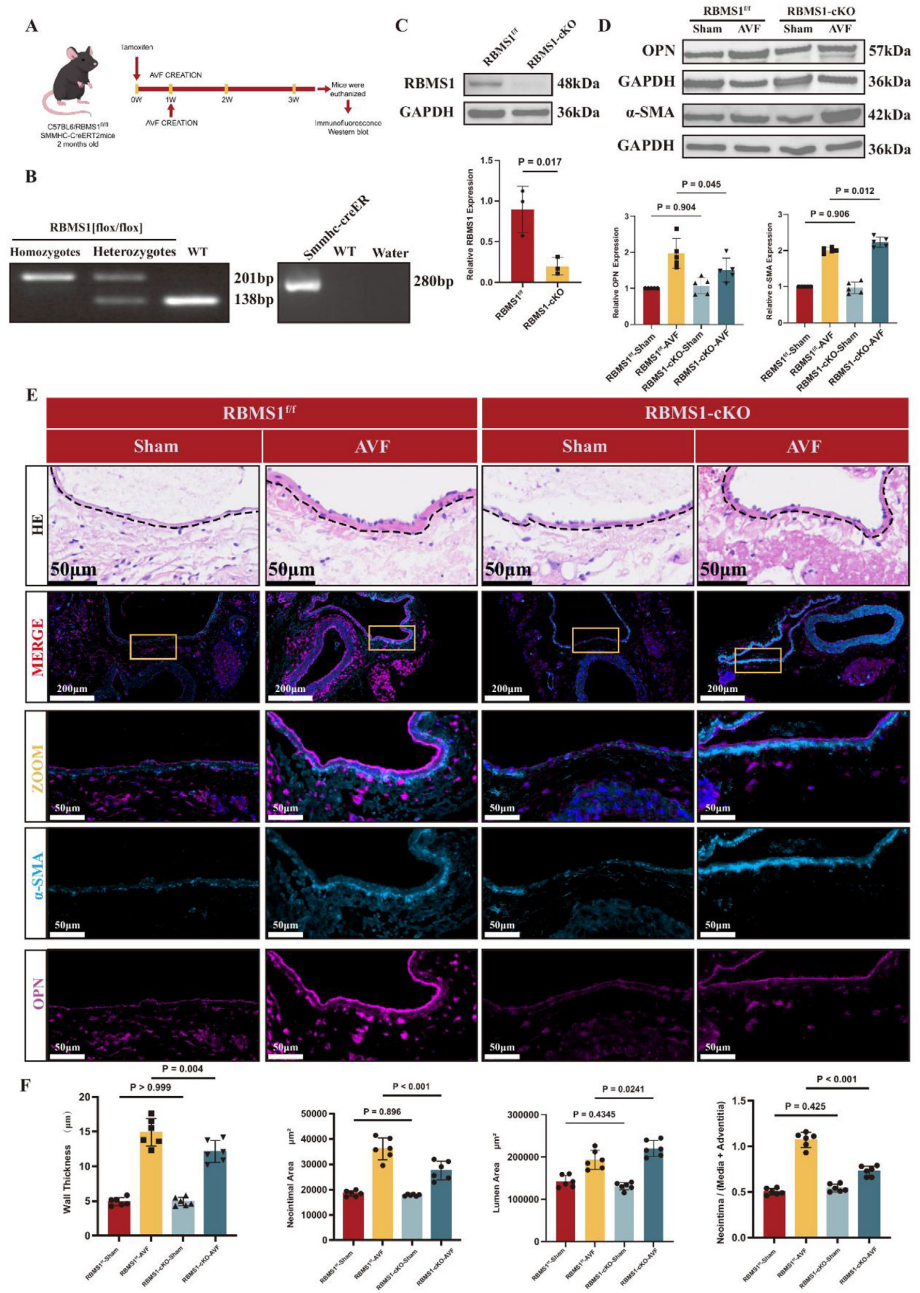
Conditional deletion of RBMS1 attenuates AVF‐associated neointimal hyperplasia. (A) Schematic of the RBMS1‐cKO mouse model. (B) Genotyping results of transgenic mice. (C) Representative Western blot images showing RBMS1 protein expression, with quantification of its relative levels (n = 3). (D) Representative Western blot images showing OPN and α‐SMA protein expression, with quantification of their relative levels (n = 5). (E) HE staining showing neointimal morphology in RBMS1‐cKO and RBMS1^f/f^ mice under sham and AVF conditions (n = 6; original magnification ×40; scale bar = 50 µm). Immunofluorescence staining showing differences in α‐SMA and OPN expression between the two groups (n = 6; scale bar = 50 µm). (F) Morphometric analysis of neointimal hyperplasia in RBMS1‐cKO and RBMS1^f/f^ mice with or without AVF creation (n = 6 per group). n represents biologically independent samples. Data are presented as mean ± SEM. Statistical significance was determined using Student's *t*‐test (C) or two‐sided one‐way ANOVA followed by Tukey's multiple comparison test (D, F).

Histological assessment and neointimal thickness measurements revealed no significant difference in intimal thickness between RBMS1‐cKO and RBMS1^f/f^ mice in the sham group. (Figure 3E,F). However, following AVF creation, RBMS1‐cKO mice exhibited significantly attenuated NIH (Figure 3E,F). Immunofluorescence and Western blot analyses further demonstrated that RBMS1 deletion mitigated VSMC phenotypic switching after AVF establishment, as evidenced by increased α‐SMA and decreased OPN expression (Figure 3D,E). These results indicate that RBMS1 deletion suppresses VSMC dedifferentiation and attenuates neointimal formation in the venous outflow segment after AVF creation.

### 3.4 RBMS1 Regulates the Generation of the MPZL1‐Δ5 Splicing Isoform in VSMCs

To investigate whether RBMS1 regulates AS in VSMCs, cytoplasmic and nuclear fractions were isolated from VSMCs. In PDGF‐BB‐induced phenotypic switching experiments, Western blot analysis revealed a marked increase in RBMS1 expression in the nuclear fraction of dedifferentiated VSMCs, while its cytoplasmic levels remained unchanged (Figure 4A). Consistent with this, immunofluorescence staining showed that RBMS1 was predominantly localized in the nucleus of VSMCs (Figure 4B), suggesting that RBMS1 may regulate AS within the nucleus.

**FIGURE 4:**
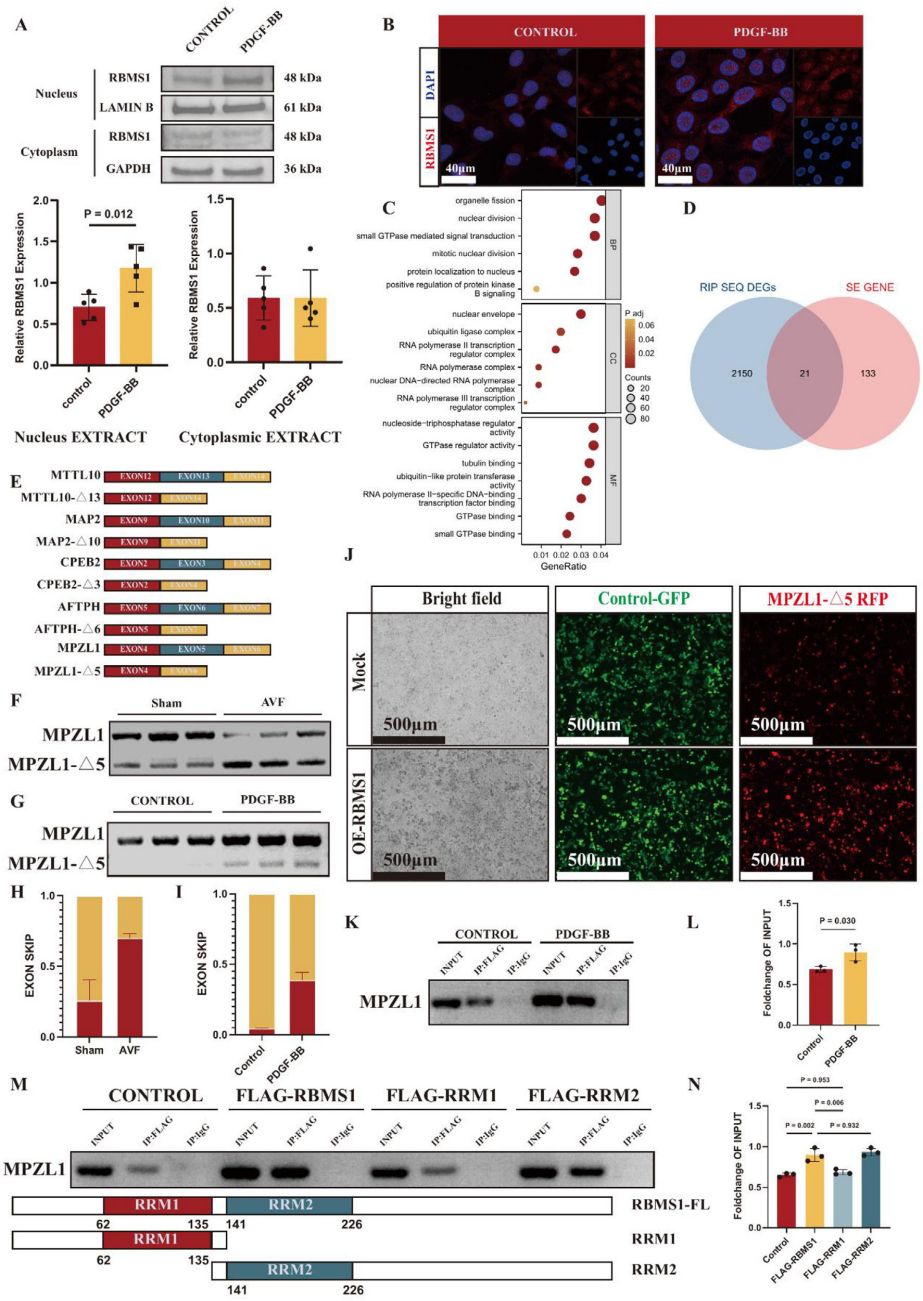
RBMS1 regulates the formation of the MPZL1‐Δ5 splice isoform through its RRM2 domain. (A) Representative Western blot images showing the protein expression levels of RBMS1 in the nucleus and cytoplasm, along with quantification of its relative expression (n = 5). (B) Immunofluorescence staining showing the subcellular localization of RBMS1 during PDGF‐BB‐induced phenotypic switching of vascular smooth muscle cells (n = 6 per group); scale bar = 40 µm. (C) Bubble plot of enrichment analysis for differentially expressed genes identified by RIP‐seq. (D) Venn diagram showing the overlap between RIP‐seq‐derived differentially expressed genes and exon‐skipping events involved in AVF remodeling. (E) Schematic representation of the top five exon‐skipping events with the largest ΔPSI values. (F) Representative agarose gel electrophoresis images showing the expression levels of the MPZL1‐Δ5 splice isoform in sham and AVF mouse tissues. (G) Representative agarose gel electrophoresis images showing the expression levels of the MPZL1‐Δ5 splice isoform during PDGF‐BB‐induced phenotypic switching of vascular smooth muscle cells. (H) Proportion of exon‐skipping events in sham and AVF mouse tissues (n = 3). (I) Proportion of exon‐skipping events during PDGF‐BB‐induced vascular smooth muscle cell phenotypic switching (n = 3). (J) Schematic diagram of the minigene reporter system used to assess MPZL1 exon 5 splicing. RFP channel fluorescence represents exon 5 splicing efficiency in HEK293T cells transfected with an empty vector or RBMS1 plasmid, while GFP channel fluorescence serves as a transfection control. (K–L) RIP‐PCR analysis of RBMS1 enrichment on MPZL1 RNA in the presence or absence of PDGF‐BB stimulation (n = 3). (M–N) RIP‐PCR analysis showing the enrichment of MPZL1 RNA by Flag‐RBMS1 wild‐type and mutant constructs (n = 3).

To identify RNA targets associated with RBMS1, RIP‐seq was performed in control VSMCs and RBMS1‐overexpressing cells, followed by enrichment analysis of differentially bound genes. The results indicated that these genes were predominantly enriched in biological processes such as organelle fission, nuclear division, small GTPase‐mediated signal transduction, mitotic nuclear division, and protein localization to the nucleus (Figure 4C).

Next, the RIP‐seq‐identified genes were intersected with ES events observed during AVF formation. The top five ES events with the most significant changes in percent spliced‐in (PSI) values were selected for validation (Figure 4D,E and Figure S5A). Among these, the MPZL1‐Δ5 splicing isoform was significantly upregulated in AVF tissues compared to sham controls (Figure 4F,H). Similarly, during PDGF‐BB‐induced VSMC phenotypic switching, the level of MPZL1‐Δ5 was also notably elevated (Figure 4G,I).

To directly validate the AS of MPZL1 pre‐mRNA, a minigene reporter system containing MPZL1 exon 5 was constructed. In this system, skipping exon 5 preserves the open reading frame (ORF) of red fluorescent protein (RFP), while inclusion of exon 5 disrupts the ORF, leading to suppressed RFP expression. Compared to the control group, RBMS1 overexpression significantly enhanced RFP fluorescence, indicating that RBMS1 promotes exon 5 skipping of MPZL1 (Figure 4J).

To explore the potential interaction between RBMS1 and MPZL1 mRNA, molecular docking analysis using AlphaFold3 was conducted. The predicted model suggested that RBMS1 could form a stable binding conformation with MPZL1 mRNA. Specifically, the RNA‐binding domains of RBMS1 exhibited favorable spatial complementarity with certain regions of MPZL1 mRNA, forming a defined interaction interface, which supports the potential for direct RBMS1–MPZL1 mRNA interaction (Figure S5B).

This interaction was further confirmed by RNA immunoprecipitation PCR (RIP‐PCR), which demonstrated that RBMS1 directly binds to MPZL1 mRNA (Figure 4K,L). To determine which RNA‐recognition motif mediates this interaction, full‐length RBMS1 and FLAG‐tagged RRM1‐ or RRM2‐containing truncation constructs were generated. RIP‐PCR showed that the RRM2‐containing construct, but not the RRM1‐containing construct, preserved MPZL1 mRNA enrichment comparable to full‐length RBMS1 (Figure 4M,N), indicating that RRM2 is the principal domain mediating RBMS1 binding to MPZL1 mRNA.

### 3.5 RBMS1 Regulates VSMC Phenotypic Responses Through MPZL1‐Δ5

To further investigate the function of FLAG‐MPZL1‐Δ5 in VSMCs, an overexpression plasmid was constructed and validated. Following transfection into VSMCs, MPZL1‐Δ5 mRNA levels were significantly elevated (Figure S6A). CCK‐8 assays demonstrated that MPZL1‐Δ5 overexpression notably promoted VSMC proliferation (Figure S6E). Consistently, wound healing and Transwell assays revealed that MPZL1‐Δ5 overexpression significantly enhanced VSMC migratory capacity (Figure S6B,D).

Additionally, increased MPZL1‐Δ5 expression in VSMCs further promoted phenotypic switching, as evidenced by persistent downregulation of contractile markers α‐SMA and SM22α and a marked upregulation of the dedifferentiation marker OPN (Figure S6C).

To examine whether RBMS1 induces VSMC phenotypic switching via MPZL1‐Δ5, VSMCs were treated with PDGF‐BB and divided into four groups. CCK‐8 assays showed that MPZL1‐Δ5 overexpression significantly rescued the impaired VSMC proliferation caused by RBMS1 knockdown (Figure S6F). Wound healing and Transwell assays confirmed that MPZL1‐Δ5 overexpression markedly reversed the reduced migratory capacity induced by RBMS1 silencing (Figure S6G,H).

Furthermore, MPZL1‐Δ5 overexpression partially corrected phenotypic alterations induced by RBMS1 knockdown. Compared to the si‐RBMS1 and si‐RBMS1 + OE‐NC groups, protein levels of contractile markers α‐SMA and SM22α were further decreased, while OPN expression was increased (Figure S6I).

These results suggest that MPZL1‐Δ5 supports VSMC proliferative, migratory, and phenotypic responses, and that its overexpression partially rescues the functional inhibition caused by RBMS1 knockdown. These findings support MPZL1‐Δ5 as a downstream mediator of RBMS1‐mediated regulation of VSMC phenotype.

### 3.6 AAV‐mediated MPZL1‐Δ5 Overexpression Exacerbates AVF Neointimal Hyperplasia in cKO‐RBMS1 Mice

To evaluate the impact of MPZL1‐Δ5 on AVF‐associated NIH in mice, a smooth muscle cell‐specific MPZL1‐Δ5 overexpression model was created using AAV. HE staining and neointimal thickness measurements were performed on venous outflow segments from mice following AVF creation. The results showed that venous NIH was markedly exacerbated in AAV‐MPZL1‐Δ5 mice compared to controls (Figure 5A,D).

**FIGURE 5:**
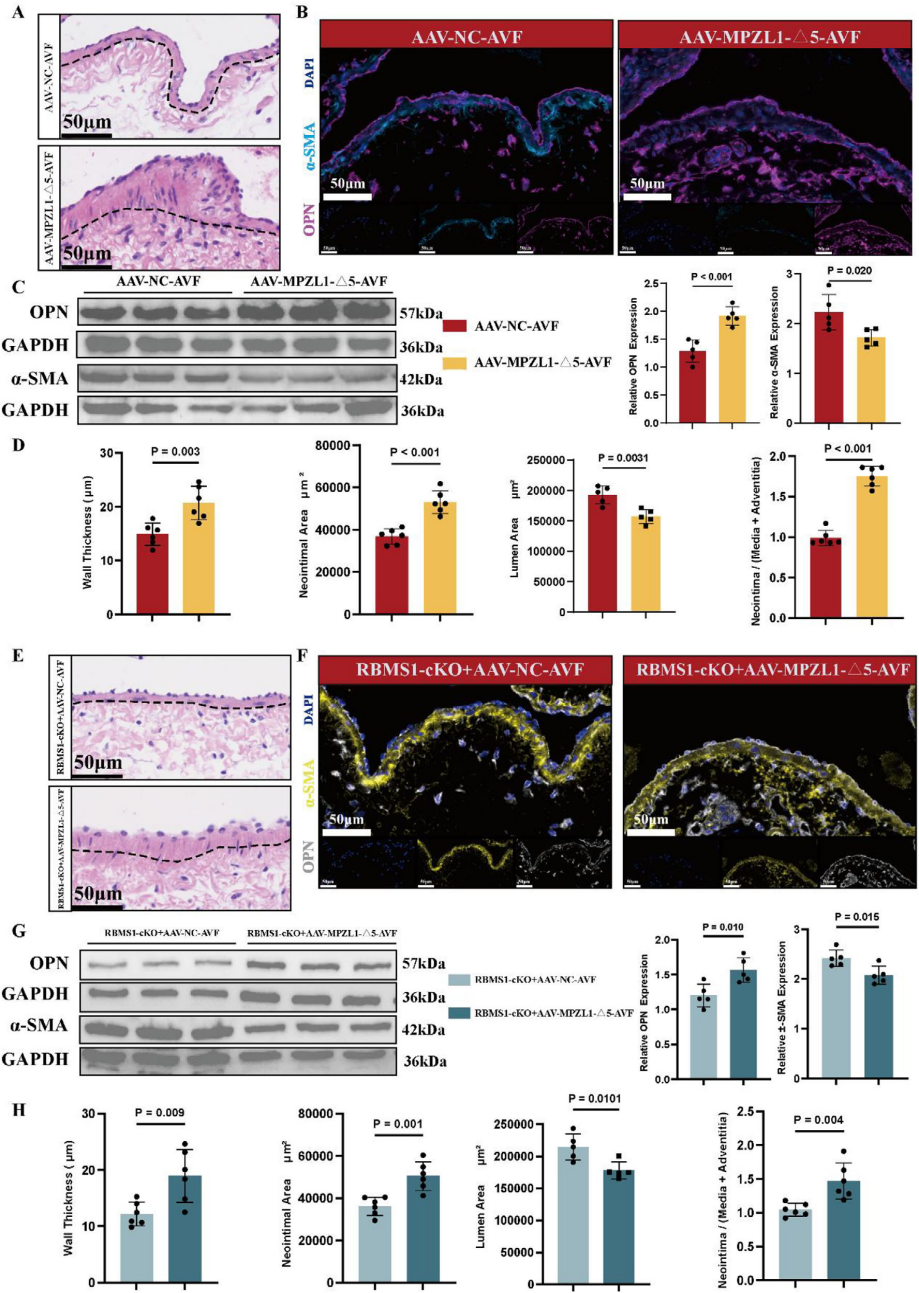
Overexpression of MPZL1‐Δ5 exacerbates AVF‐associated neointimal hyperplasia. (A) HE staining showing neointimal morphology in the AAV‐MPZL1‐Δ5 and AAV‐NC groups under AVF conditions (n = 6; original magnification ×40; scale bar = 50 µm). (B) Immunofluorescence staining showing differences in α‐SMA and OPN expression between the two groups (n = 6; scale bar = 50 µm). (C) Representative Western blot images showing OPN and α‐SMA protein expression, with quantification of relative expression levels (n = 5). (D) Morphometric analysis of neointimal hyperplasia in the venous outflow segment from AAV‐MPZL1‐Δ5 and AAV‐NC mice following AVF creation (n = 6 per group). (E) HE staining showing neointimal morphology in the AAV‐MPZL1‐Δ5 and AAV‐NC groups in cKO‐RBMS1 mice under AVF conditions (n = 6; original magnification ×40; scale bar = 50 µm). (F) Immunofluorescence staining showing differences in α‐SMA and OPN expression between the two groups in cKO‐RBMS1 mice (n = 6; scale bar = 50 µm). (G) Representative Western blot images showing OPN and α‐SMA protein expression, with quantification of relative expression levels (n = 5). (H) Morphometric analysis of neointimal hyperplasia in veins from the AAV‐MPZL1‐Δ5 and AAV‐NC groups in cKO‐RBMS1 mice following AVF creation (n = 6 per group). n represents biologically independent samples. Data are presented as mean ± SEM. Statistical significance was determined using Student's *t*‐test (C, D, G, H).

Next, changes in VSMC phenotypic markers were assessed. Immunofluorescence staining and Western blot analyses revealed that MPZL1‐Δ5 overexpression further promoted VSMC phenotypic switching after AVF creation, as indicated by downregulation of α‐SMA and upregulation of OPN (Figure 5B,C).

To assess whether RBMS1 promotes AVF‐associated NIH through induction of MPZL1‐Δ5, cKO‐RBMS1 mice were divided into two groups and transduced with either AAV‐NC or AAV‐MPZL1‐Δ5. HE staining and neointimal thickness measurements of venous outflow segments from cKO‐RBMS1 mice after AVF creation demonstrated that MPZL1‐Δ5 overexpression significantly aggravated venous NIH (Figure 5E,H).

Consistently, analysis of VSMC phenotypic markers revealed that in cKO‐RBMS1 mice after AVF creation, MPZL1‐Δ5 overexpression further enhanced VSMC phenotypic switching, evidenced by decreased α‐SMA and increased OPN expression, as shown by immunofluorescence staining and Western blot analyses (Figure 5F,G).

These observations indicate that smooth muscle cell‐specific overexpression of MPZL1‐Δ5 is associated with exacerbated NIH and enhanced VSMC phenotypic switching in murine AVF models. Moreover, upregulation of MPZL1‐Δ5 in the RBMS1 cKO background is sufficient to further promote these pathological changes, supporting a functional contribution of MPZL1‐Δ5 to RBMS1‐associated AVF remodeling in this experimental context.

### 3.7 MPZL1‐Δ5 Interacts with G3BP1, and G3BP1 Knockdown Suppresses VSMC Proliferative, Migratory, and Phenotypic Responses

Previous analyses suggested that RBMS1 may be involved in regulating small GTPase‐mediated signaling pathways, with G3BP1 identified as a key regulatory factor. To explore the interaction between the MPZL1‐Δ5 splice isoform and G3BP1, co‐immunoprecipitation assays were conducted in cells transfected with the FLAG‐MPZL1‐Δ5 plasmid. The results confirmed that FLAG‐MPZL1‐Δ5 physically interacts with G3BP1 (Figure 6A), indicating that RBMS1 might promote VSMC phenotypic switching by modulating the interaction between MPZL1‐Δ5 and G3BP1.

**FIGURE 6:**
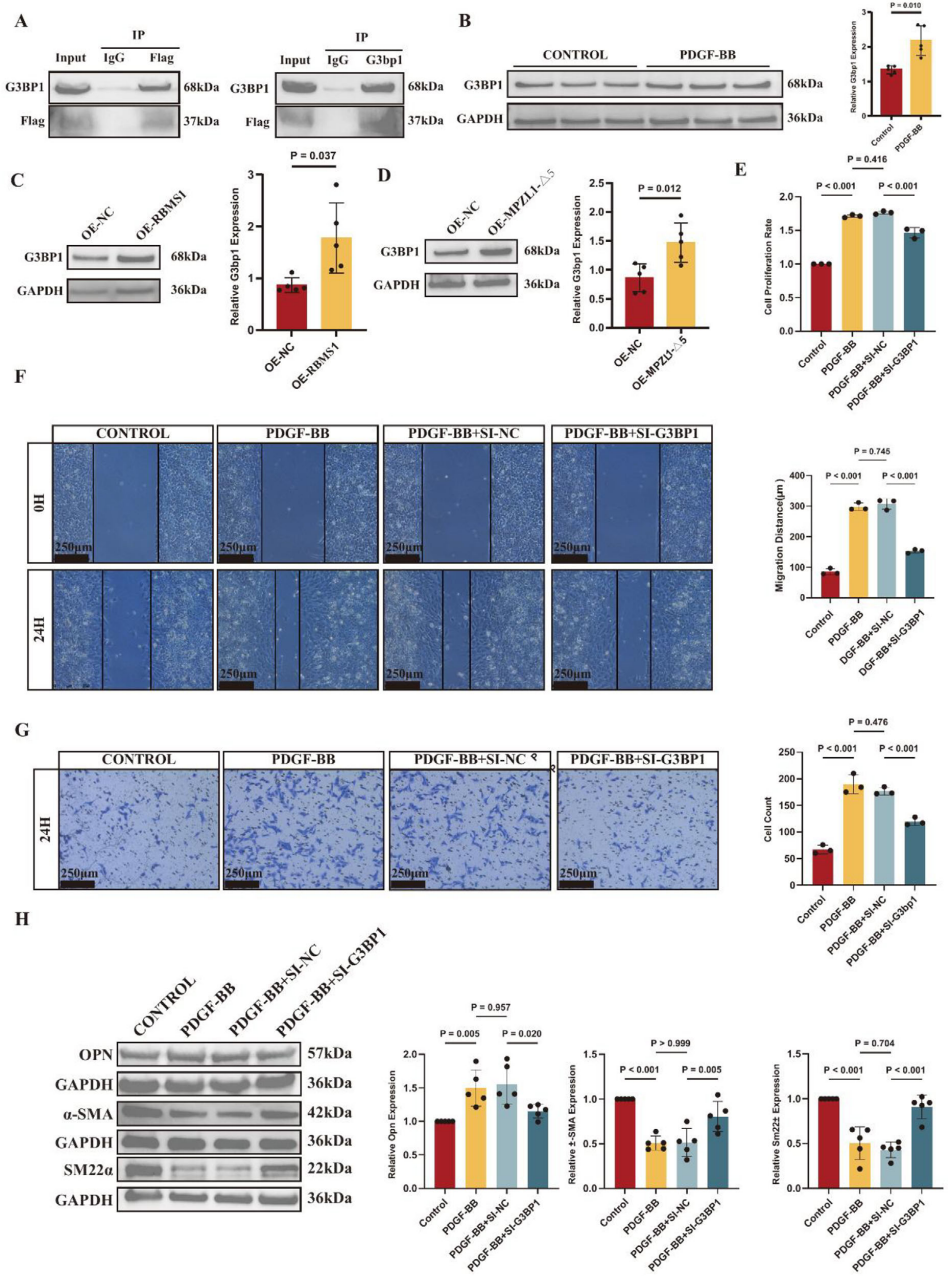
Knockdown of G3BP1 inhibits VSMC proliferation, migration, and phenotypic switching. (A) Co‐immunoprecipitation (Co‐IP) assays were performed to evaluate the interaction between MPZL1‐Δ5 and G3BP1. (B) Representative Western blot images showing the protein expression of G3BP1 in PDGF‐BB‐induced dedifferentiated VSMCs and the corresponding quantitative analysis (n = 5). (C) Representative Western blot images showing the protein expression of G3BP1 in VSMCs overexpressing RBMS1 and the corresponding quantitative analysis (n = 5). (D) Representative Western blot images showing the protein expression of G3BP1 in VSMCs overexpressing MPZL1‐Δ5 and the corresponding quantitative analysis (n = 5). (E) Cell proliferation and viability of VSMCs were assessed using the CCK‐8 assay (n = 3). (F–G) VSMC migratory capacity was evaluated using Transwell and wound‐healing (scratch) assays; scale bars = 250 µm for both assays (n = 3). (H) Representative Western blot images showing the expression of phenotypic marker proteins OPN, α‐SMA, and SM22α and the corresponding quantitative analysis (n = 5). n indicates the number of biologically independent samples. Data are presented as mean ± SEM. Statistical significance was determined using Student's *t*‐test or Welch's corrected *t*‐test (B–D), and one‐way ANOVA followed by Tukey's multiple‐comparison test (E–H). NC indicates negative control; si indicates small interfering RNA.

In a PDGF‐BB‐induced VSMC phenotypic switching model, G3BP1 expression was significantly upregulated in dedifferentiated VSMCs (Figure 6B). Western blot analysis further revealed that overexpression of either RBMS1 or MPZL1‐Δ5 resulted in elevated G3BP1 protein levels (Figure 6C,D).

Subsequently, a siRNA‐mediated G3BP1 knockdown system was established and validated. VSMCs were categorized into four groups. Functional assays demonstrated that, under PDGF‐BB stimulation, si‐G3BP1 significantly inhibited VSMC migration, as shown by both wound healing and Transwell assays (Figure 6F,G). Additionally, CCK‐8 assays indicated that G3BP1 knockdown substantially reduced VSMC proliferation (Figure 6E).

To confirm successful dedifferentiation induction, decreased expression of contractile markers α‐SMA and SM22α, alongside increased OPN expression, was observed. Importantly, G3BP1 knockdown in dedifferentiated VSMCs partially reversed these phenotypic changes, as evidenced by a partial restoration of α‐SMA and SM22α expression and suppression of OPN upregulation (Figure 6H).

These results suggest that G3BP1 contributes to VSMC phenotypic marker changes and functional responses, supporting its role as a downstream effector of MPZL1‐Δ5‐mediated signaling.

### 3.8 RBMS1 And MPZL1‐Δ5 Regulate G3BP1‐Associated AKT Activation During VSMC Phenotypic Responses

To further assess whether RBMS1 promotes VSMC phenotypic switching via regulation of G3BP1, VSMCs were divided into four groups. Wound healing and Transwell assays showed that silencing G3BP1 significantly attenuated the migratory capacity enhanced by RBMS1 overexpression (Figure 7A,B).

**FIGURE 7:**
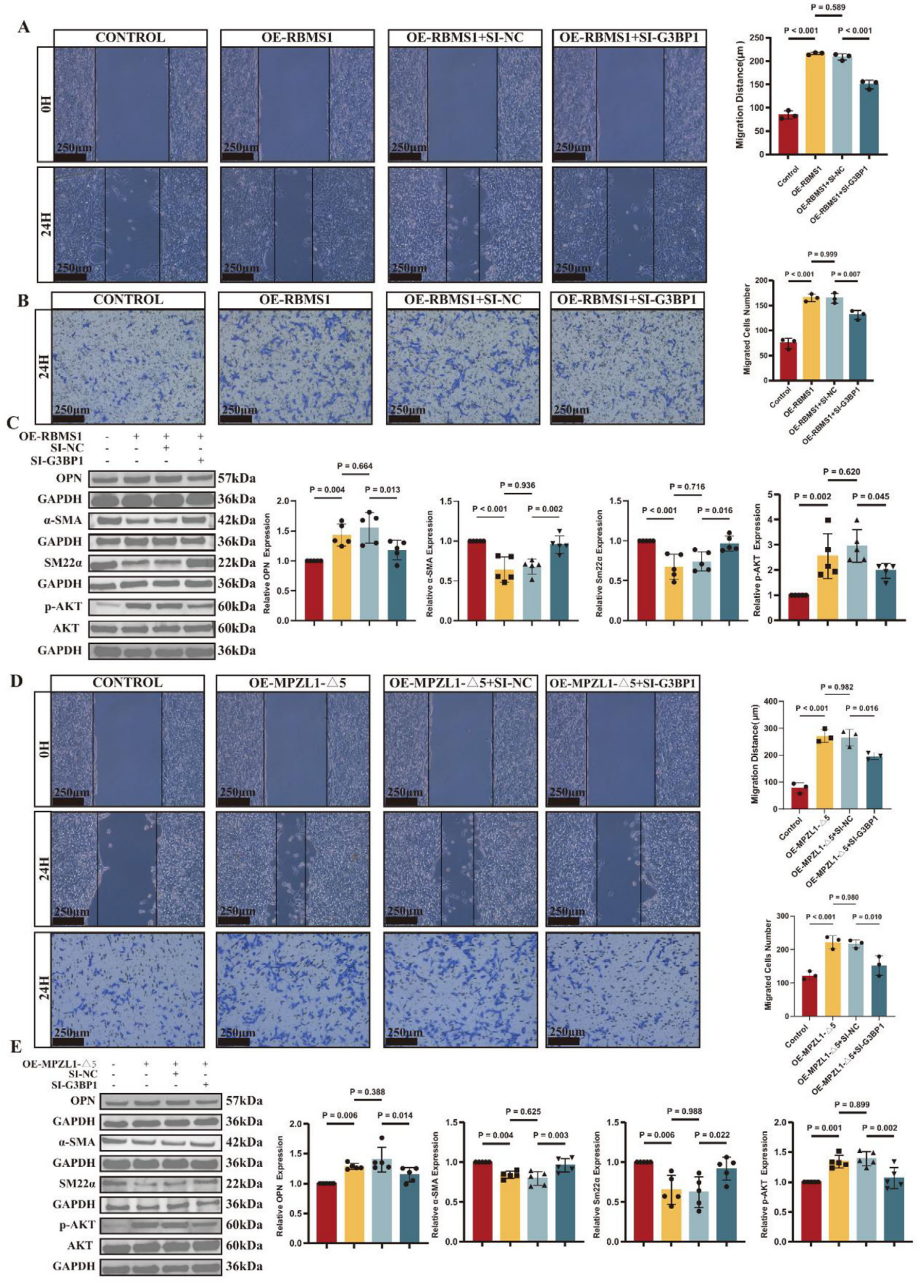
Knockdown of G3BP1 suppresses MPZL1‐Δ5‐ and RBMS1 overexpression‐induced smooth muscle phenotypic switching and AKT activation. (A–B) VSMC migratory capacity was evaluated using Transwell and wound‐healing assays; scale bar = 250 µm for Transwell assays and 250 µm for wound‐healing assays (n = 3). (C) Representative Western blot images showing the expression of phenotypic marker proteins OPN, α‐SMA, SM22α, and AKT activation, along with quantitative analysis (n = 5). (D) VSMC migratory capacity was evaluated using Transwell and wound‐healing assays; scale bar = 250 µm for Transwell assays and 250 µm for wound‐healing assays (n = 3). (E) Representative Western blot images showing the expression of phenotypic marker proteins OPN, α‐SMA, and SM22α, along with quantitative analysis (n = 5). n indicates biologically independent replicates. Data are presented as mean ± SEM. Statistical analyses were performed using one‐way ANOVA followed by Tukey's multiple‐comparison test (A–E). NC indicates negative control; si indicates small interfering RNA.

Western blot analyses revealed that G3BP1 inhibition markedly reversed the phenotypic switching induced by RBMS1 overexpression. Compared to the OE‐RBMS1 and OE‐RBMS1 + si‐NC groups, protein levels of the contractile markers α‐SMA and SM22α were significantly elevated, while expression of the dedifferentiation marker OPN was substantially reduced following G3BP1 knockdown (Figure 7C).

Previous studies have indicated that G3BP1 activates the AKT signaling pathway [32]. Based on this, this study investigated whether RBMS1‐regulated G3BP1 affects AKT activation. Western blot analysis showed significantly higher phosphorylated AKT (p‐AKT) levels in the OE‐RBMS1 and OE‐RBMS1 + si‐NC groups compared to controls, indicating that RBMS1 overexpression activates the AKT pathway. Conversely, G3BP1 knockdown markedly suppressed RBMS1‐induced AKT phosphorylation (Figure 7C). These results suggest that RBMS1 promotes VSMC phenotypic switching through activation of the G3BP1‐mediated AKT signaling pathway.

Given that RBMS1 regulates VSMC phenotypic switching through MPZL1‐Δ5 modulation, this study further explored whether MPZL1‐Δ5 is associated with phenotypic modulation via G3BP1 regulation. VSMCs were divided into four groups. Wound healing and Transwell assays revealed that G3BP1 silencing significantly diminished the migratory capacity induced by MPZL1‐Δ5 overexpression (Figure 7D).

Western blot analysis confirmed that G3BP1 inhibition significantly reversed MPZL1‐Δ5‐induced phenotypic switching. Compared to the OE‐MPZL1‐Δ5 and OE‐MPZL1‐Δ5 + si‐NC groups, protein levels of α‐SMA and SM22α were significantly increased, while OPN expression was markedly reduced upon G3BP1 knockdown (Figure 7E).

Next, this study assessed whether MPZL1‐Δ5 influences AKT activation via G3BP1. The results showed that p‐AKT levels were significantly elevated in the OE‐MPZL1‐Δ5 and OE‐MPZL1‐Δ5 + si‐NC groups compared to controls, suggesting that MPZL1‐Δ5 overexpression activates the AKT pathway. Notably, G3BP1 silencing considerably suppressed MPZL1‐Δ5‐induced AKT phosphorylation (Figure 7E).

These results suggest that RBMS1‐associated VSMC phenotypic responses are linked to G3BP1‐mediated AKT activation.

## 4 Discussion

In this study, we identified RBMS1 as an important regulator of VSMC‐associated neointimal remodeling in AVFs. To position this mechanism within the human AVF cellular landscape, we analyzed the cell‐type distribution of RBMS1 expression in a recently published single‐cell atlas [7]. The atlas contained diverse vascular, stromal, and immune populations, while the VSMC group showed the highest within‐cell‐type RBMS1‐positive fraction among the nine major cell populations. This distribution was consistent with spatial transcriptomic and human tissue evidence showing increased RBMS1 signals in α‐SMA‐positive vascular regions. RBMS1 expression was also increased in PDGF‐BB‐treated VSMCs. Together, these findings provided a human‐data‐supported basis for investigating the function of RBMS1 in VSMC‐associated neointimal remodeling.

Functionally, smooth muscle‐targeted RBMS1 overexpression exacerbated neointimal formation, whereas conditional RBMS1 deletion attenuated it in murine AVFs. In vitro, RBMS1 promoted VSMC proliferation, migration, and phenotypic modulation. Mechanistically, RBMS1 promoted MPZL1 exon 5 skipping to generate the MPZL1‐Δ5 isoform. MPZL1‐Δ5 interacted with G3BP1 and enhanced AKT activation, thereby mediating RBMS1‐dependent VSMC responses. These findings define the RBMS1–MPZL1‐Δ5–G3BP1–AKT pathway as an important VSMC‐associated mechanism contributing to NIH during AVF remodeling.

Much of the current understanding of VSMC phenotypic plasticity originates from studies of arterial injury and atherosclerosis. The venous outflow segment after AVF creation, however, is exposed to distinct hemodynamic forces and cellular interactions. Our findings extend the post‐transcriptional regulation of VSMC plasticity to venous neointimal remodeling and identify RBMS1‐mediated MPZL1 splicing as a mechanism contributing to AVF‐associated NIH (Figure 8).

**FIGURE 8:**
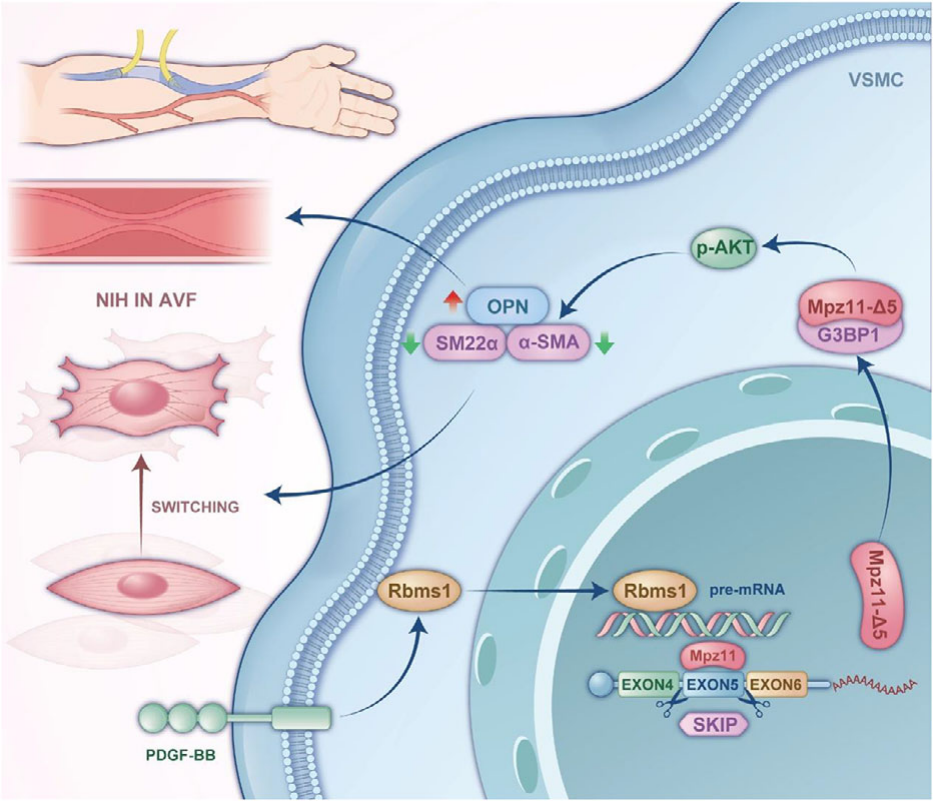
Proposed VSMC‐associated mechanism of RBMS1‐mediated neointimal remodeling in arteriovenous fistula.

The datasets and specimens analyzed in this study represent distinct temporal and clinical contexts. GSE244317, collected on postoperative day 7, and our day‐14 murine AVF model were used to investigate early postoperative neointimal remodeling; neither was designed to model early maturation failure. In contrast, GSE283628 and our institutional postoperative specimens were obtained after kidney transplantation or during later clinically indicated surgical procedures and therefore reflect postoperative or chronic remodeling rather than the early biological events governing maturation. GSE305423 and GSE220796 provided clinically stratified mature and failed AVF data, but RBMS1 upregulation was observed in both outcome groups. We therefore distinguish early postoperative remodeling from early maturation failure, late stenosis of a previously functional access, chronic remodeling, and AVF removal after kidney transplantation.

Interpretation of the mouse aortocaval puncture model also requires consideration of its specific anatomical and hemodynamic configuration. This model creates a reproducible, high‐flow central AVF between the aorta and inferior vena cava, with disturbed and potentially bidirectional venous outflow. Its vessel size, anatomical location, fistula geometry, and flow environment differ from those of upper‐extremity human AVFs. Nevertheless, the model provides a technically reproducible platform with needle‐controlled fistula geometry and limited suture‐related injury, and it shares features with the side‐to‐side configuration of emerging endovascular AVF procedures. It is therefore useful for studying early venous outflow remodeling, disturbed flow, thrombus formation, and neointimal responses. However, the model should not be considered equivalent to a human upper‐extremity AVF, and the neointimal lesions observed in this setting should not be interpreted as evidence of clinical stenosis, access dysfunction, or AVF failure. In the present study, neointimal measurements are interpreted as a localized structural component of early experimental AVF remodeling.

Within these boundaries, the human findings remain descriptive. The small and unpaired institutional cohort did not permit patient‐level correlations of RBMS1 or MPZL1‐Δ5 expression with quantitative histological or functional outcomes. Accordingly, the human analyses establish that RBMS1 expression changes during postoperative AVF remodeling, whereas the mechanistic experiments specifically support a role for the RBMS1–MPZL1‐Δ5 pathway in VSMC‐associated neointimal remodeling during the early postoperative interval.

Although our findings identify RBMS1 as a regulator of neointimal remodeling, the clinical significance of NIH should be considered within the broader remodeling response. Histological studies have shown that intimal hyperplasia is common in pre‐access veins and that neither pre‐existing nor postoperative intimal thickening consistently predicts AVF maturation or primary unassisted patency [4, 5]. In human cohorts, postoperative stenosis and medial fibrosis have shown stronger associations with nonmaturation than intimal expansion alone. The effect of NIH on access function is therefore shaped by concurrent outward remodeling, lumen expansion, wall fibrosis, vascular compliance, inflammation, and thrombosis [33]. Accordingly, our in vivo findings support a role for RBMS1 in regulating the neointimal component of early AVF remodeling, identifying a specific pathological mechanism that contributes to venous wall remodeling after AVF creation.

In the cardiovascular system, VSMCs typically maintain a mature contractile phenotype under physiological conditions to regulate vascular tone and ensure hemodynamic stability [34]. However, during pathological conditions such as vascular injury, atherosclerotic plaque formation, and NIH, VSMCs undergo extensive phenotypic switching, transitioning to a synthetic phenotype with enhanced proliferative and migratory capabilities. This transformation is governed by a complex gene regulatory network, integrating both transcriptional and post‐transcriptional mechanisms [35].

Among various post‐transcriptional regulatory mechanisms, AS stands out as a rapid and reversible mode of regulation that enables precise modulation of protein structure and function, either during development or in response to environmental stress By producing multiple protein isoforms from a single gene, without expanding genome size, AS significantly enhances proteomic diversity and functional complexity [5]. Growing evidence highlights the essential roles of distinct AS events and their corresponding splicing regulators in modulating VSMC phenotypic plasticity. Several splice variants have been implicated in regulating VSMC proliferation and migration [36, 37]. However, comprehensive studies of AS events and their functional relevance during autologous AVF remodeling remain scarce.

To fill this gap, this study employed the rMATS analytical framework to systematically identify AS events in a murine AVF remodeling model. Our findings revealed that ES was the predominant type of AS event, suggesting that exon skipping may be an important splicing event associated with early AVF remodeling. Therefore, understanding the regulatory mechanisms underlying ES and its impact on VSMC phenotypic switching is crucial for unraveling the molecular basis of AVF‐associated NIH [38].

AS is regulated by multiple factors, including chromatin structure, RNA polymerase II elongation rate, and the activity of core splicing machinery. However, RBPs, whose expression is often tissue‐specific, play a pivotal role in orchestrating selective AS programs [39]. Through motif enrichment analysis, this study identified RBMS1 as a key RBP involved in AVF remodeling and subsequently validated its relevance in both human and murine tissues.

RBMS1, a member of the RNA‐binding motif protein family, possesses two nucleic acid‐binding domains, each containing highly conserved submotifs [38, 40]. This structural characteristic enables RBMS1 to regulate AS of mRNA [22]. Previous studies have demonstrated that RBMS1 specifically binds to single‐stranded and double‐stranded DNA within the replication origin sequences of the c‐Myc gene, thus participating in DNA replication, transcriptional regulation, and cellular transformation [41]. Notably, RBMS1 overexpression has been shown to suppress α‐SMA promoter activity in visceral smooth muscle, with elevated expression observed in spontaneous atherosclerosis models, suggesting a potential role in smooth muscle phenotypic modulation [40].

In this study, smooth muscle cell‐specific overexpression of RBMS1 via AAV exacerbated NIH in murine AVFs, while genetic deletion of RBMS1 attenuated neointimal formation. In vitro, plasmid‐mediated RBMS1 overexpression enhanced VSMC proliferation, migration, and phenotypic switching, while siRNA‐mediated knockdown produced the opposite effects. Notably, RBMS1 was predominantly localized in the nucleus of VSMCs, supporting its role as a splicing factor that regulates AS‐dependent phenotypic switching, thereby promoting AVF‐associated NIH.

To explore the relationship between RBMS1 and VSMC phenotypic switching, RIP‐seq was performed to identify mRNAs bound by RBMS1. Integrative analyses using Venn diagrams, volcano plots, and GO enrichment identified several AS targets associated with AVF remodeling, including the exon 5‐deleted isoform of MPZL1, which was significantly upregulated in both AVF tissues and dedifferentiated VSMCs.

MPZL1, a type I transmembrane glycoprotein, has been identified as a substrate of Src homology 2 domain‐containing protein tyrosine phosphatase 2 (SHP2), a key regulator of several signaling pathways, including PI3K–AKT, Janus kinase–signal transducer and activator of transcription (JAK–STAT), and Src family kinase pathways [42, 43]. MPZL1 has been widely implicated in tumorigenesis, particularly in cell adhesion and migration, with its functions appearing evolutionarily conserved [44, 45, 46, 47]. Aberrant expression or amplification of MPZL1 is associated with invasive and metastatic behaviors in various malignancies. The exon 5‐included isoform of MPZL1 has been described in glioma as an oncogenic isoform that binds SHP2 and activates downstream PI3K–AKT and extracellular signal‐regulated kinase (ERK) signaling, driving cancer stem cell proliferation, whereas exon 5 skipping attenuates these effects [48]. These apparently divergent effects may reflect differences in cell type and disease context.

This study identified MPZL1 as a candidate downstream target of RBMS1 and showed that RBMS1 promotes the generation of the exon 5‐deleted isoform during AVF remodeling. Smooth muscle cell‐specific overexpression of this isoform aggravated NIH and enhanced VSMC phenotypic switching in vivo and in vitro. Moreover, MPZL1‐Δ5 overexpression further increased NIH in RBMS1‐deficient mice. Together, these findings support a functional contribution of MPZL1‐Δ5 to RBMS1‐associated VSMC responses and neointimal remodeling in experimental AVFs.

G3BP1 is a multifunctional regulator of GTPase signaling, primarily localized in the cytoplasm, but it can also translocate to the nucleus. Emerging evidence highlights its diverse biological roles, including regulation of cell proliferation, migration, apoptosis, differentiation, and RNA metabolism [49, 50]. In cardiovascular biology, G3BP1 has been shown to participate in vascular remodeling, inflammation, stress responses, and cardiac hypertrophy, and it plays a key role in activating the AKT signaling pathway across various disease contexts [51].

In this study, co‐immunoprecipitation experiments revealed a direct interaction between the exon 5‐deleted isoform of MPZL1‐Δ5 and G3BP1 in VSMCs. Overexpression of RBMS1 or MPZL1‐Δ5 significantly increased G3BP1 expression, whereas G3BP1 knockdown inhibited AKT activation and reversed the phenotypic switching of VSMCs induced by the overexpression of either upstream regulator. These findings suggest that the RBMS1–MPZL1‐Δ5 axis promotes AVF‐associated NIH by enhancing G3BP1 expression and activating the AKT signaling pathway.

This study has several limitations. First, the in vivo experiments used a mouse aortocaval puncture model, and tissues were collected 14 days after AVF creation. This model and time point were selected to examine early venous outflow remodeling and neointimal hyperplasia rather than flow‐limiting stenosis, AVF failure, or long‐term patency [52]. The findings should therefore be interpreted as evidence that RBMS1 contributes to the neointimal component of early AVF remodeling, rather than as evidence of altered clinical maturation or access outcomes. Second, the in vivo experiments were conducted in animals without CKD, whereas clinical AVFs are created predominantly in patients with advanced CKD or ESKD. CKD alters endothelial integrity, inflammation, vascular calcification, VSMC behavior, and extracellular matrix remodeling, and experimental studies have shown that it accelerates neointimal formation and endothelial barrier dysfunction in murine AVFs [53]. Thus, whether CKD modifies RBMS1‐dependent responses requires validation in CKD AVF models. Third, only male mice were included. Because sex differences may influence AVF remodeling and access outcomes, studies involving both sexes are required to determine whether RBMS1 has sex‐specific effects [54, 55]. Fourth, the institutional human cohort was small and unpaired, limiting statistical power and generalizability and precluding patient‐level correlations between RBMS1 or MPZL1‐Δ5 expression and quantitative histological or functional outcomes. Fifth, the functional experiments focused on VSMCs and did not determine how RBMS1‐dependent VSMC responses interact with stromal, immune, or endothelial signaling within the multicellular AVF environment. Further studies integrating lineage‐resolved analyses and cell–cell communication approaches will be required to characterize these interactions.

In conclusion, our findings support the RBMS1–MPZL1‐Δ5–G3BP1–AKT axis as a VSMC‐associated signaling mechanism that contributes to early experimental AVF neointimal remodeling. These results advance the mechanistic understanding of post‐transcriptional regulation of VSMC phenotypic responses during the early postoperative remodeling of AVFs.

## Author Contributions

**Zongda Li**: conceptualization, investigation, Writing – original draft, visualization, formal analysis, methodology. **Chengwei Zhang**: methodology. **Xinying Yu**: methodology. **Shuang Yao**: conceptualization, methodology, writing – review and editing. **Yiting Lai**: methodology. **Xiaozhu Sun**: methodology, validation. **Yao Zhao**: supervision, project administration, funding acquisition. **Xiaoyu Lan**: methodology. **Tingting Hu**: methodology, software. **Wenjin Li**: methodology. **Zhenzhen Ma**: methodology. **Huimin Ma**: methodology, data curation. **Zihan Wang**: methodology. **Haihai Liang**: writing – review and editing, supervision, project administration, investigation, conceptualization. **Jundong Jiao**: conceptualization, investigation, writing – review and editing, funding acquisition, supervision, resources.

## Funding

This research was supported by grants from the National Natural Science Foundation of China (Nos. 82170756 and 82500915), the Key Research and Development Program of Heilongjiang Province (No. 2023ZX06C06), and the Science and Technology Innovation Base Award Program of Heilongjiang Province (No. JD24D006).

## Ethics Approval and Consent to Participate

The animal experiments were approved by the institutional animal ethics committee of the Second Affiliated Hospital of Harbin Medical University (approval no. YJSDW2024‐131). The research involving human participants was approved by the Institutional Review Board of the Second Affiliated Hospital of Harbin Medical University (approval no. YJSKY2024‐081). Written informed consent was obtained from all participants before surgery.

## Conflicts of Interest

The authors declare no conflicts of interest.

## Supporting information




**Supporting File**: advs78286‐sup‐0001‐SuppMat.docx.

## Data Availability

The data that support the findings of this study are available from the corresponding author upon reasonable request.
