# Supplementary material for: RBMS1 Contributes to Early Arteriovenous Fistula Neointimal Remodeling Through Modulation of VSMC Phenotypic Responses
Source: Adv Sci (Weinh). 2026 Oct 11:e78286. Online ahead of print. doi: 10.1002/advs.78286 (PMC13655268; doi:10.1002/advs.78286)
Supplement: Supplementary file 1 [file ADVS-9999-e78286-s001.docx]

**Supplemental Methods**

**Differential Gene Expression and Spatial Transcriptomic Analysis**

**Single-cell RNA sequencing data were processed using a standard analysis pipeline.**

Low-quality cells were excluded during quality control, followed by normalization and logarithmic transformation of the expression matrix. Highly variable genes were identified, and principal component analysis (PCA) was performed. Using significant principal components, a cell–cell adjacency graph was constructed, followed by unsupervised clustering. Uniform Manifold Approximation and Projection (UMAP) was applied for dimensionality reduction and visualization. Cell types were annotated based on canonical marker gene expression patterns and prior literature. Spatial transcriptomic data underwent quality control and normalization using comparable procedures. Spatial feature plots were created to visualize the expression of key genes across tissue sections.

**Alternative Splicing Analysis**

AS events were identified and quantitatively analyzed using replicate Multivariate Analysis of Transcript Splicing (rMATS) software (version 4.1.1). This software employed a statistical modeling framework specifically designed to detect differential splicing events between two or more RNA sequencing sample groups. Differential splicing events were identified by calculating the statistical significance for each splicing event and evaluating changes in the percent spliced-in (PSI) between groups. Five major types of AS events were analyzed, including exon skipping , alternative 3′ splice sites, alternative 5′ splice sites, mutually exclusive exons, and intron retention. Differential AS events were considered significant with a false discovery rate threshold of less than 0.05 and an absolute change in PSI of at least 0.1.

**Cell Migration**

For wound healing assays, VSMCs were seeded into culture inserts (ibidi, Germany). After 24 hours, the inserts were removed, and images were captured at 0 and 24 hours using a Leica microscope to assess wound closure. For Transwell migration assays, VSMCs suspended in 200 μL of serum-free DMEM were seeded into the upper chambers of 24-well Transwell inserts. The lower chambers were filled with 600 μL of DMEM containing 10% FBS. After 24 hours, the migrated cells on the lower surface of the membrane were fixed with 4% PFA and stained with crystal violet. Cells remaining on the upper surface were gently removed using a cotton swab, and images of the migrated cells were acquired using a Leica microscope.

**Histology and Immunohistochemistry**

Tissue samples were fixed in paraformaldehyde at 4°C for 24 hours, followed by graded ethanol dehydration, xylene clearing, and paraffin embedding. Paraffin sections, 5 µm in thickness, were prepared. For histological analysis, sections were deparaffinized, rehydrated, and stained with hematoxylin and eosin (HE), followed by dehydration, clearing, and mounting for microscopic examination. For histomorphometric analysis, the venous outflow segment of the inferior vena cava was sampled from a predefined region cranial to and adjacent to the fistula, rather than from the fistula opening itself. The corresponding anatomical level was sampled in sham-operated animals. Tissues were fixed, paraffin-embedded, and sectioned transversely at approximately 5 μm thickness. Sections were collected at 50 μm intervals, and three sections were analyzed for each animal. Neointimal and vessel-wall areas were delineated using predefined anatomical boundaries and consistent criteria across experimental groups. Measurements from the three sections were averaged to generate a single animal-level value for statistical analysis. Images were quantified using ImageJ. For the revised analysis, image files were randomly coded by an investigator who was not involved in quantification, and group information was concealed until all measurements had been completed and checked. Additional longitudinal sections were used for anatomical visualization only and were not treated as independent observations.

For immunohistochemistry, deparaffinized and rehydrated sections underwent heat-induced antigen retrieval in citrate buffer. Endogenous peroxidase activity was blocked, followed by nonspecific protein blocking. Sections were incubated with primary antibodies overnight at 4°C and subsequently with enzyme-labeled secondary antibodies. Signal detection was performed using diaminobenzidine, and nuclei were counterstained with hematoxylin. For immunohistochemistry, sections were incubated with anti-RBMS1 antibody (1:100, Cat# 11061-2-AP, ProteinTech, China).

**Immunofluorescence**

Paraffin sections were deparaffinized, rehydrated, and subjected to antigen retrieval using citrate buffer. Following blocking, sections were incubated with primary antibodies overnight at 4°C, followed by incubation with fluorescently labeled secondary antibodies. Nuclear staining was performed using 4′,6-diamidino-2-phenylindole (DAPI) (Cat# P0131, Beyotime, China), and sections were mounted with antifade medium prior to fluorescence microscopy. For cellular immunofluorescence, cells grown on coverslips were fixed, permeabilized, and blocked before incubation with primary and fluorescent secondary antibodies. Nuclei were counterstained, and samples were examined under a fluorescence microscope. For immunofluorescence staining, the following primary antibodies were used: anti-RBMS1 antibody (1:100, Cat# 11061-2-AP, ProteinTech, China), anti-α-SMA antibody (1:100, Cat# 14395-1-AP, ProteinTech, China), and anti-OPN antibody (1:100, Cat# ab63856, Abcam, UK).

**Quantitative Gene Expression Analysis**

Total RNA was extracted from tissues or cells using a phenol–chloroform-based method (Cat#ET101-01-V2, TransGen Biotech, China). Complementary DNA was synthesized using a reverse transcription kit (Cat#R323-01, Vazyme, China). Quantitative gene expression analysis was performed using a fluorescence-based detection system(Cat#R323-01, TransGen Biotech, China). Gene expression levels were normalized to glyceraldehyde-3-phosphate dehydrogenase (GAPDH), and relative expression levels were calculated using the comparative threshold cycle (ΔΔCT) method. Primer sequences are listed in Supplementary Table 2.

**Protein Analysis by Western blot**

Proteins were extracted using radioimmunoprecipitation assay buffer (Cat#P0013B, Beyotime, China). Equal amounts of protein were separated by sodium dodecyl sulfate–polyacrylamide gel electrophoresis (SDS-PAGE) and transferred onto polyvinylidene fluoride (PVDF) membranes. Membranes were incubated with primary antibodies, followed by fluorescent secondary antibodies, and signals were detected using an imaging system. The following primary antibodies were used: anti-RBMS1 antibody (1:1000, Cat# 11061-2-AP, ProteinTech, China), anti- OPN antibody (1:1000, Cat# ab63856, Abcam, UK), anti-α-SMA antibody (1:1000, Cat# 14395-1-AP, ProteinTech, China), anti-GAPDH antibody (1:10000, Cat# 60004-1-Ig, ProteinTech, China), anti-SM22α antibody (1:2000, Cat# 10493-1-AP, ProteinTech, China), anti-DYKDDDDK tag antibody (1:2000, Cat# 66008-4-Ig, ProteinTech, China), anti-SNAIL (1:1000, Cat# 340942, ZenBio, China), anti-SLUG (1:1000, Cat# 213286, Biodragon, China), anti-G3BP1 antibody (1:1000, Cat# A3968, ABclonal, China), anti-phospho-Akt (Ser473) antibody (1:2000, Cat# 4060, Cell Signaling Technology, USA), and anti-Akt (pan) antibody (1:2000, Cat# 4691, Cell Signaling Technology, USA).

**RNA-Binding Protein Immunoprecipitation Assay (RIP)**

VSMCs were lysed for 10 minutes using cell lysis buffer supplemented with protease and RNase inhibitors (P0102, Geneseed, China). The lysates were incubated overnight at 4°C with magnetic beads conjugated to either RBMS1 antibody (RBMS1 group) or control IgG (IgG group). After immunoprecipitation, RNA was extracted using a spin-column-based method, reverse-transcribed into cDNA, and analyzed by PCR.

**Agarose Gel Electrophoresis**

For agarose gel electrophoresis, a 1.5% gel was prepared by dissolving agarose powder in TAE electrophoresis buffer, then heating it in a microwave until fully dissolved. After cooling to 50–60°C, Gel-Red (10 µL per 100 mL, 1:10,000 dilution, Cat#D0139, Beyotime, China) was added, and the solution was poured into a casting tray with combs inserted. Once solidified, the gel was placed in an electrophoresis tank filled with TAE buffer. Samples were loaded into the wells, and electrophoresis was carried out at 120 V for 40 minutes until the bromophenol blue dye front approached the bottom of the gel. DNA bands were visualized under UV light, and images were captured using a gel documentation system.
